# Effect of different application duration of a venous foot pump on prevention of venous thromboembolism after hip and knee arthroplasty: a multicenter prospective clinical trial

**DOI:** 10.1186/s12891-023-06921-w

**Published:** 2023-12-01

**Authors:** Siqi Gu, Yu-e Chen, Mingxing Lei, Jiahui Li, Wanying Li, Meihong Zhang, Hongxia Zhu, Mengying Ma, Dan Kong, Yuan Gao

**Affiliations:** 1 Department of Orthopaedics, The First Medical Center of Chinese PLA General Hospital, Beijing, 100853 China; 2 Department of Orthopaedics, The Fourth Medical Center of Chinese PLA General Hospital, Beijing, 100048 China; 3 Department of Orthopaedics, The First Affiliated Hospital of Soochow University, Jiangsu, 215006 China; 4 Department of Orthopaedics, Hainan Hospital, PLA General Hospital, Hainan, 572013 China; 5 Nursing Department, The First Medical Center of Chinese PLA General, Beijing, 100853 China

**Keywords:** Hip arthroplasty, Knee arthroplasty, Venous foot pump, Venous thromboembolism

## Abstract

**Objective:**

To investigate the optimal duration of applying a venous foot pump (VFP) in the prevention of venous thromboembolism (VTE) following hip and knee arthroplasty.

**Methods:**

A total of 230 patients undergoing hip and knee arthroplasty between March 2021 and March 2022 in orthopaedic departments of four major teaching hospitals were prospectively enrolled. Patients were randomly divided into five groups based on the duration of the VFP application. Postoperative deep vein thromboses (DVT), including proximal, distal, and intermuscular DVT, were recorded for analysis. Postoperative blood coagulation examinations, such as D-dimer and active partial thromboplastin time (APTT), pain outcome, and degree of comfort were also collected.

**Results:**

Two of the 230 patients withdrew due to early discharge from the hospital, and 228 patients were included in the final analysis. The mean age was 60.38 ± 13.33 years. The baseline characteristics were comparable among the five groups. Compared with the other groups, patients treated with 6-hour VFP had the lowest incidence of DVT (8.7%, 4/46), followed by those treated with 1-hour VFP (15.2%, 7/46), 12-hour VFP (15.6%, 7/45), 18-hour VFP(17.8%, 8/45) and 20-hour VFP(21.7%, 10/46), but with no significant difference (*P* = 0.539). Regarding postoperative blood coagulation examinations, patients treated with 6-hour VFP had the lowest D-dimer (*P* = 0.658) and the highest APTT (*P* = 0.262) compared with the other four groups. 6-hour VFP also had the lowest pain score (*P* = 0.206) and the highest comfort score (*P* = 0.288) compared with the other four groups.

**Conclusions:**

Six hours may be the optimal duration of applying VFP for the prevention of VTE in patients undergoing hip and knee arthroplasty in terms of VTE incidence, postoperative blood coagulation examinations, pain outcomes, and comfort scores.

**Supplementary Information:**

The online version contains supplementary material available at 10.1186/s12891-023-06921-w.

## Background

Venous thromboembolism (VTE) is a serious health issue worldwide. It refers to the abnormal coagulation of blood in the vein. VTE is one of the most important complications after orthopaedic surgery [1]. It mainly includes deep vein thrombosis (DVT) and pulmonary embolism (PE). Globally, there are nearly 10 million cases of VTE annually. VTE is a substantial contributor to the global burden of disease [2]. Evidence has shown that the incidence of VTE is 60.0% after major trauma surgery, 42–57% among patients treated with a total hip replacement, and 41–85% among patients treated with a total knee replacement [3]. In addition, with growing populations and longer life expectancy, the prevalence of this disease is increasing. The annual incidence of acute VTE is 1–2 cases per 1,000 population, which increases exponentially with age in both men and women [4]. It is reported that approximately 300,000 people die of VTE every year in the United States [5]. In addition to the increase in the medical burden to society, VTE can also lead to long-term disability, reduce the quality of life, and threaten the lives of patients. VTE-related healthcare costs are estimated to be $ 3.3 billion in Europe and $ 10 billion in the United States [6, 7]. Thus, a better understanding of VTE is urgent to develop appropriate preventive strategies for VTE.

Currently, the preventive strategies for VTE mainly include pharmacologic and mechanical thromboprophylaxis among patients treated with knee and/or hip replacement. Pharmacologic thromboprophylaxis included direct or indirect thrombin inhibitors, vitamin K antagonists, and factor Xa inhibitors. Mechanical thromboprophylaxis includes graduated compression stockings, intermittent pneumatic compression, venous foot pumps (VFPs), and transcutaneous electrical nerve stimulation [8]. Among these approaches, VFP is a promising method of thromboprophylaxis and is characterized by pulsed pneumatic therapy for the plantar foot by simulating the human physiological foot pump [9]. However, there is no consensus on the optimal duration of applying VFP to prevent VTE events in patients undergoing knee and hip orthopaedic surgery. Whereas some studies have shown that one-hour VFP can prevent VTE, other studies have reported that 6-hour and 20-hour VFP can also reduce the incidence of VTE [10, 11]. Nonetheless, these studies were either small sample size analyses or retrospective studies. The Chinese expert consensus has recommended the use of intermittent pneumatic compression (IPC) for more than 18 h per day. However, there needs to be high-quality evidence to support this proposition.

Therefore, the present study aimed to investigate the optimal duration of applying VFP for the prevention of VTE in patients receiving hip and knee arthroplasty. A multicenter prospective clinical trial was conducted to generate high-quality evidence.

## Patients and methods

### Patients and study design

**Fig. 1:**
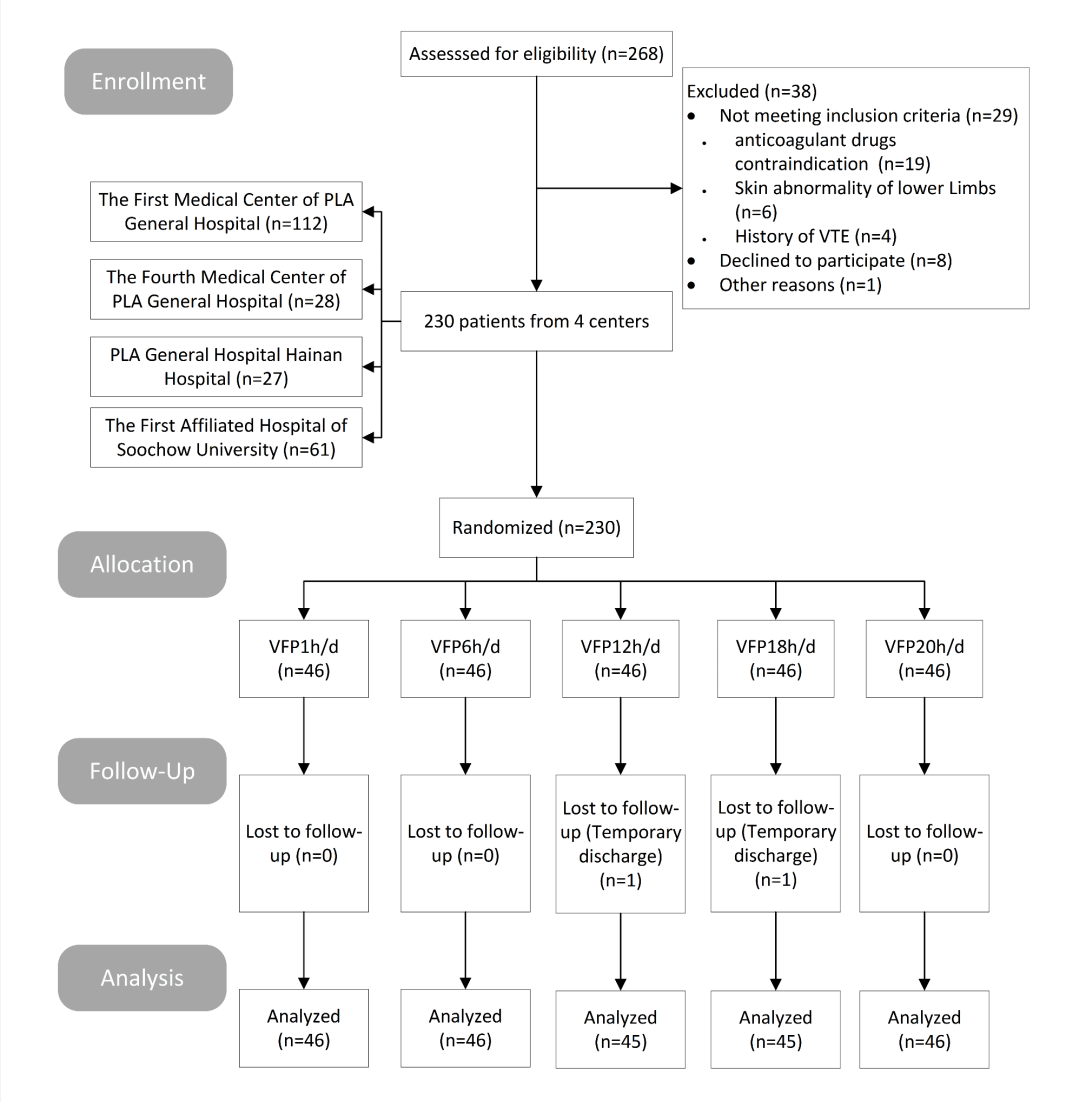
A flowchart of patient enrolment process

A total of 230 patients undergoing hip and knee arthroplasty between March 2021 and March 2022 in orthopaedic departments of four major teaching hospitals, including the First Medical Center of Chinese PLA General Hospital, the Fourth Medical Center of Chinese PLA General Hospital, Hainan Hospital of Chinese PLA General Hospital, and the First Affiliated Hospital of Suzhou University, were prospectively enrolled. The inclusion criteria were as follows: (1) patients receiving hip and/or knee arthroplasty, (2) aged ≥ 18 years, (3) had a Caprini score of ≥ 3 points [12], and (4) were conscious and aware of self-identity, space, time, and expressing well-being. The exclusion criteria were as follows: (1) patients previously diagnosed with VTE, (2) unable to receive drug anticoagulation due to severe cardiac, liver, and/or kidney dysfunction, and severe organ bleeding tendency, (3) allergic to VFP equipment materials, and (4) reluctance to continue participating in the study. The criteria for withdrawal were as follows: (1) changes in condition, temporary discharge or transfer; (2) withdrawal due to personal wishes; (3) The test should be stopped immediately when the skin at the application site is red, swollen, and damaged; (4) The requirement of daily use time was not met. The flow chart of patient selection is displayed in Fig. 1.

All patients voluntarily participated in the study and written informed consent was obtained from each patient. This study was approved by the Medical Ethics Committee of Chinese PLA General Hospital (No. 2021-002-01) and the First Affiliated Hospital of Suzhou University (No. 2,021,053). We registered our study in the Chinese Clinical Trial Registry (02/01/2022) (ChiCTR2200055166). Data and materials are available upon reasonable request with the permission of the corresponding authors.

According to the expert consensus on mechanical prevention of VTE in China, the longest recommended time was ≥ 18 h per day. A previous study pointed out that 6–9 h per day could also be effective in the prevention of VTE [13]. In other studies, 30 min to 1 h per day was found to be sufficient [14]. Therefore, after thorough consideration, the duration of the VFP application was finally divided into five groups: 1, 6, 12, 18, and 20 h per day.

### Sample calculation

The study referred to the formula of multiple independent sample rates: n = $\frac{{1641.6 \times \lambda }}{{({{\sin }^{ - 1}} \times \sqrt {P\max } - {{\sin }^{ - 1}} \times \sqrt {P{{\min }^2}} }};$ λ is 11.94 when α = 0.05 and β = 0.20. According to the literature [15, 16], when $Pmax$ = 20.0% and $Pmin$ = 0.65%, then a sample size of n = 41 is obtained. Considering that the sample drop-out rate was 10%, at least 46 cases were enrolled in each group, and at least 230 cases overall.

### Randomization and blinding

Patients were randomly divided into five groups by computer random method based on the duration of the VFP application. SPSS26.0 software was used to generate a random sequence, and the random assignment scheme was saved in light-tight envelopes. Envelopes were opened sequentially in the order of enrollment, and patients were assigned to the assigned group according to the assignment scheme in the envelope. Four centers enrolled participants at the same time. Enrollment was terminated when the total number of participants reached the final required sample size. Because patients and study operators were aware of device use, the study was blinded only to study personnel who performed the ultrasonography.

### Interventions

**Fig. 2:**
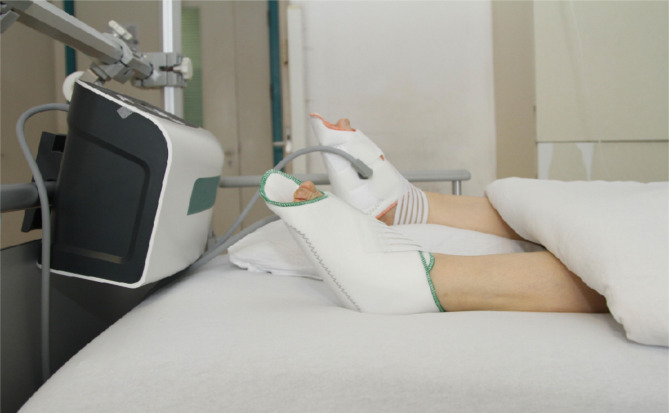
The foot venous pump worn by the patient

All patients received pharmacologic and mechanical thromboprophylaxis [5]. The safety and efficacy of low molecular weight heparin have been demonstrated. [17] Patients were administered an anticoagulant agent (dalteparin, 5000 IU per day) from the first day after surgery to discharge. Regarding mechanical thromboprophylaxis, all patients were treated with bilateral lower extremity VFP (CWHEAL, CWH-8000). The pressure value was set as 130 mmHg, and the inflation and pressurization durations were 3 and 20 s, respectively. All patients in each group were applied continuously except for getting out of bed. If the application was suspended midway, each application should not be less than 30 min, and the interval time should not exceed 10 min. According to the group of patients, the daily application time was set as follows: group A 1 h/d, group B 6 h/d, group C 12 h/d, group D 18 h/d, and group E 20 h/d. The VFP worn by the patient is shown in Fig. 2.

### Primary outcome

The primary outcome was postoperative DVT, including proximal DVTs such as thromboses in the popliteal vein, femoral vein, or iliac vein, distal DVTs such as thromboses in the anterior tibial vein, posterior tibial vein, or peroneal vein, and intermuscular DVT. DVT was examined using colour Doppler flow imaging at postoperative day 3. The diagnostic criteria of DVT were: (1) dilated vena cava, (2) substantial echo, and (3) intraluminal filling defect. If the symptoms of PE were observed in patients, pulmonary arteriography was applied.

### Secondary outcome

The secondary outcome included postoperative blood coagulation examinations, such as D-dimer and active partial thromboplastin time (APTT), pain outcome, and the degree of comfort. D-dimer and APTT were recorded on postoperative days 1 and 3. The visual analog scale (VAS) [18] was used to assess pain outcome, and a 100-point scale was used to assess the degree of comfort, with a higher score indicating greater comfort when using the VFP.

### Variables and definitions

Patient’s baseline characteristics included (1) demographics such as age, sex, nationality, body mass index (BMI), temperature, heart rate, respiration rate, systolic blood pressure (SBP), diastolic blood pressure (DBP), blood type, hypertension, and diabetes, (2) surgery-related information such as the type of surgery, type of anesthesia, blood loss, operation time, catheterization, blood transfusion, perioperative use of hemostatics, drainage, and perioperative use of the hormone, and (3) preoperative laboratory examinations such as thrombin time (TT), APTT, prothrombin time (PT), D-dimer, fibrin, hemoglobin, platelet count, and erythrocyte sedimentation rate (ESR). The patient’s temperature, heart rate, respiration rate, SBP, and DBP were collected on the first day after hospitalization. Operation time was defined as the time from skin incision to skin closure.

### Quality control

A series of measures were considered to guarantee the quality of the study. Firstly, all investigators were trained on how to evaluate patients using the Caprini thrombus risk assessment scale, and all patients received the same education on thrombus prevention, including signs and symptoms of VTE and bleeding, the importance of seeking help if symptoms develop, and conservative measures to prevent VTE such as ambulation and avoiding dehydration. Secondly, an ultrasound physician with the same qualification was designated to complete the ultrasound examination and diagnosis of VTE for enrolled patients in each center. In addition, ultrasound physicians were blinded to the patient’s categories. Thirdly, Each center designated a responsible nurse as a researcher in the center to collect the data. The unified data collection form and the same type of measuring instrument were used to ensure the compliance of data collection and reduce measurement bias. The data were regularly recorded and summarized, and quality control was monitored and audited at any time to ensure the integrity and accuracy of all data.

### Statistical analysis

Categorical variables were expressed as proportion and continuous variables as the mean ± standard deviation. The chi-square test was utilized to evaluate differences between categorical variables and analysis of variance (ANOVA) was used to compare the means between three or more groups for continuous variables. Repeated measures ANOVA was used to analyze D-dimer and APTT. If sphericity was violated (Mauchly’s Test of Sphericity), Greenhouse-Geisser correction was applied. Multivariate analysis was used to screen potential risk factors for predicting DVT, and the area under the receiver operating characteristic (AUROC) curve was used to evaluate the predictive performance of each significant variable. All data were analyzed using SPSS (Version 26.0) or R programing language (Version 4.1.2). A P-value of less than 0.05 (two-sided) was considered statistically significant.

## Results

### Baseline characteristics

Two of the 230 patients withdrew due to early discharge from the hospital. Thus, 228 patients were included in the final analysis. The mean age was 60.38 ± 13.33 years. The majority of patients were females, accounting for 63.2% of all patients. Regarding the surgical site, the right knee accounted for 31.6%, followed by the left hip (21.1%) and right hip (20.2%). The mean BMI was 25.70 ± 4.23 kg/m^2^, indicating an overweight status. The patient’s temperature, heart rate, respiration rate, and DBP were in the normal range. Approximately 88.6% of patients received general anesthesia, 26.8% had a blood transfusion, 38.6% received hemostatic therapy, 29.8% had drainage, and 20.2% received hormone treatment. More details on blood coagulation examinations are shown in Table 1. Baseline characteristics were comparable among the five groups (Table 1).

**Table 1:** Baseline characteristics

Characteristics	Overall	Groups				
		1 h	6 h	12 h	18 h	20 h
n	228	46	46	45	45	46
Sex = male/female (%)	84/144 (36.8/63.2)	19/27 (41.3/58.7)	19/27 (41.3/58.7)	15/30 (33.3/66.7)	13/32 (28.9/71.1)	18/28 (39.1/60.9)
Age (mean (SD))	60.38 (13.22)	57.59 (15.84)	61.98 (13.22)	63.42 (13.87)	59.73 (10.89)	59.22 (11.42)
Type of surgery (%)						
Right knee arthroplasty	72 (31.6)	14 (30.4)	14 (30.4)	12 (26.7)	16 (35.6)	16 (34.8)
Left knee arthroplasty	43 (18.9)	13 (28.3)	9 (19.6)	6 (13.3)	6 (13.3)	9 (19.6)
Right and left knee arthroplasty	10 (4.4)	2 (4.3)	2 (4.3)	2 (4.4)	2 (4.4)	2 (4.3)
Right hip arthroplasty	46 (20.2)	6 (13.0)	12 (26.1)	8 (17.8)	13 (28.9)	7 (15.2)
Left hip arthroplasty	48 (21.1)	8 (17.4)	8 (17.4)	15 (33.3)	6 (13.3)	11 (23.9)
Right and left hip arthroplasty	9 (3.9)	3 (6.5)	1 (2.2)	2 (4.4)	2 (4.4)	1 (2.2)
Hypertension = no/yes (%)	171/57 (75.0/25.0)	33/13 (71.7/28.3)	35/11 (76.1/23.9)	32/13 (71.1/28.9)	32/13 (71.1/28.9)	39/7 (84.8/15.2)
Diabetes = no/yes (%)	224/4 (98.2/1.8)	46/0 (100.0/0.0)	44/2 (95.7/4.3)	45/0 (100.0/0.0)	45/0 (100.0/0.0)	44/2 (95.7/4.3)
Nationality = Han/Minority (%)	221/7 (96.9/3.1)	42/4 (91.3/8.7)	45/1 (97.8/2.2)	45/0 (100.0/0.0)	45/0 (100.0/0.0)	44/2 (95.7/4.3)
BMI (kg/m^2^, mean (SD))	25.70 (4.23)	26.10 (3.90)	24.87 (5.34)	26.14 (3.84)	26.32 (4.23)	25.10 (3.56)
Temperature (℃, mean (SD))	36.47 (0.32)	36.40 (0.29)	36.37 (0.29)	36.54 (0.31)	36.48 (0.35)	36.56 (0.33)
Heart rate (mean (SD))	77.22 (7.09)	77.87 (7.04)	75.74 (6.62)	78.40 (7.94)	77.18 (8.09)	76.96 (5.51)
Respiration rate (mean (SD))	18.05 (0.92)	18.02 (0.71)	18.26 (0.71)	18.04 (0.88)	18.00 (0.90)	17.91 (1.28)
Systolic blood pressure (mmHg, mean (SD))	136.72 (15.98)	137.83 (18.21)	137.59 (15.45)	138.44 (16.61)	132.89 (14.75)	136.83 (14.70)
Diastolic blood pressure (mmHg, mean (SD))	80.80 (9.84)	79.61 (9.74)	81.54 (11.70)	81.87 (9.28)	80.53 (8.65)	80.48 (9.80)
Blood type (%)						
A	58 (25.4)	12 (26.1)	9 (19.6)	14 (31.1)	13 (28.9)	10 (21.7)
AB	21 (9.2)	5 (10.9)	5 (10.9)	4 (8.9)	2 (4.4)	5 (10.9)
B	76 (33.3)	15 (32.6)	13 (28.3)	16 (35.6)	16 (35.6)	16 (34.8)
O	73 (32.0)	14 (30.4)	19 (41.3)	11 (24.4)	14 (31.1)	14 (30.4)
Anesthesia = General/ Epidural (%)	202/26 (88.6/11.4)	40/6 (87.0/13.0)	38/8 (82.6/17.4)	39/6 (86.7/13.3)	42/3 (93.3/6.7)	43/3 (93.5/6.5)
Blood loss (ml, mean (SD))	213.97 (168.81)	237.39 (161.43)	232.61 (127.46)	205.00 (146.42)	219.56 (210.10)	175.22 (186.44)
Operation time (min, mean (SD))	138.43 (57.97)	146.54 (54.84)	120.98 (38.13)	139.13 (59.82)	139.49 (80.63)	146.07 (46.84)
Catheterization = no/yes (%)	227/1 (99.6/0.4)	46/0 (100.0/0.0)	46/0 (100.0/0.0)	45/0 (100.0/0.0)	44/1 (97.8/2.2)	46/0 (100.0/0.0)
Blood transfusion = no/yes (%)	167/61 (73.2/26.8)	29/17 (63.0/37.0)	36/10 (78.3/21.7)	30/15 (66.7/33.3)	31/14 (68.9/31.1)	41/5 (89.1/10.9)
Perioperative use of hemostatics = no/yes (%)	140/88 (61.4/38.6)	23/23 (50.0/50.0)	33/13 (71.7/28.3)	30/15 (66.7/33.3)	26/19 (57.8/42.2)	28/18 (60.9/39.1)
Drainage = no/yes (%)	160/68 (70.2/29.8)	31/15 (67.4/32.6)	35/11 (76.1/23.9)	28/17 (62.2/37.8)	33/12 (73.3/26.7)	33/13 (71.7/28.3)
Perioperative use of hormone = no/yes (%)	182/46 (79.8/20.2)	34/12 (73.9/26.1)	33/13 (71.7/28.3)	40/5 (88.9/11.1)	35/10 (77.8/22.2)	40/6 (87.0/13.0)
TT (s, mean (SD))	16.64 (2.55)	16.49 (2.69)	15.60 (2.53)	16.67 (2.43)	16.70 (2.52)	17.73 (2.19)
APTT (s, mean (SD))	29.98 (5.24)	29.99 (4.80)	32.17 (5.28)	29.38 (6.18)	29.75 (5.17)	28.58 (4.11)
PT (s, mean (SD))	12.20 (1.49)	12.33 (1.51)	12.58 (1.47)	12.04 (1.72)	12.10 (1.47)	11.95 (1.22)
D-dimer (mg/L, mean (SD))	0.91 (2.14)	0.59 (0.82)	1.64 (4.12)	0.93 (1.25)	0.63 (0.67)	0.77 (1.64)
Fibrin (mean (SD))	3.27 (2.05)	3.05 (0.54)	3.23 (1.24)	3.34 (1.14)	3.23 (2.67)	3.53 (3.33)
Hemoglobin (mean (SD))	131.19 (15.24)	133.17 (15.56)	131.52 (16.07)	127.42 (14.09)	128.91 (15.99)	134.78 (13.79)
Platelet (mean (SD))	222.88 (64.74)	230.37 (73.90)	215.61 (60.49)	226.44 (70.00)	227.00 (61.30)	215.15 (57.92)
ESR (mean (SD))	12.39 (11.57)	11.65 (8.50)	14.39 (15.72)	14.84 (11.65)	9.76 (8.49)	11.29 (11.58)

BMI: body mass index; TT: thrombin time; APTT: active partial thromboplastin time; PT: prothrombin time; ESR: erythrocyte sedimentation rate

### Comparison of the incidence of DVT among the five groups

**Fig. 3:**
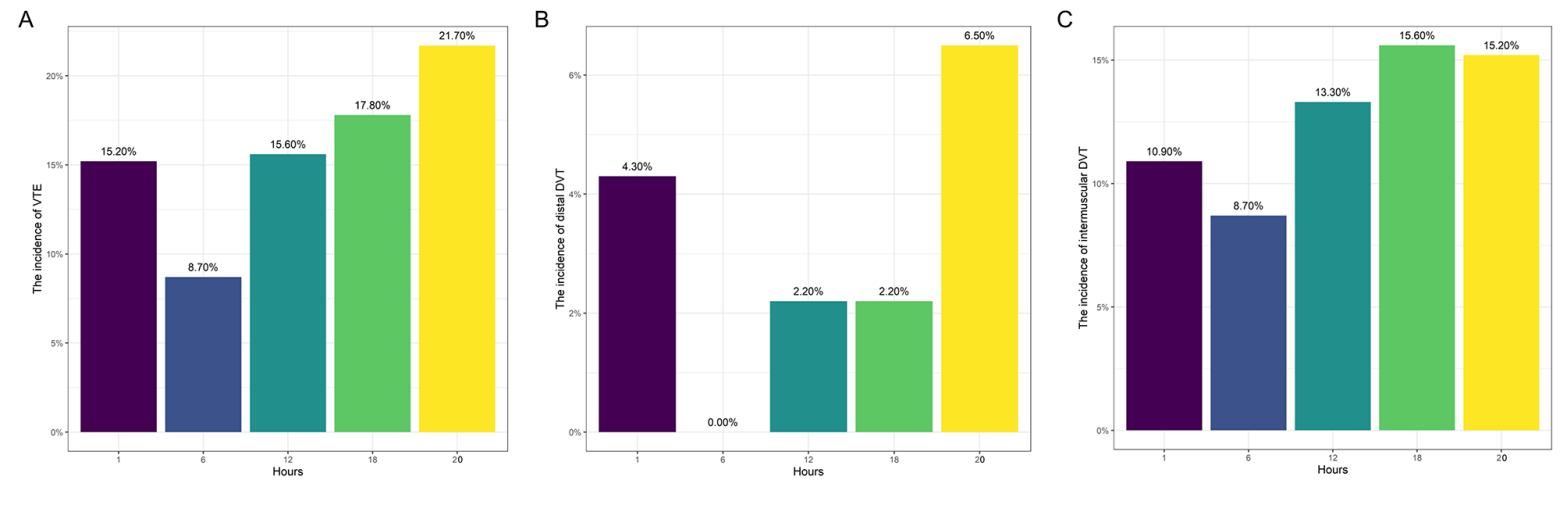
Histogram of patient groups with venous thromboembolism. **A.** The incidence of venous thromboembolism in the five groups; **B.** The incidence of distal deep vein thrombosis in the five groups; **C.** The incidence of intermuscular deep vein thrombosis in the five groups.

Compared with the other groups, patients treated with 6-hour VFP had the lowest incidence of DVT (8.7%, 4/46), followed by 1-hour VFP (15.2%, 7/46) and 12-hour VFP (15.6%, 7/45) ( *P* = 0.539, Table 2). When patients were treated with VFP for ≥ 6 h, there was an increasing trend in the incidence of DVT. The proportion of patients increased from 8.7% (4/46) after 6-hour VFP treatment to 21.7% (10/46) after > 18-hour VFP (Fig. 3A). Similar trends were also observed in terms of distal DVT (Fig. 3B) and intermuscular DVT (Fig. 3C) based on subgroup analysis. However, no proximal DVT or PE was observed.

**Table 2:** Comparison of the incidence of VTE between groups

Groups	VTE incidence	Subgroup analysis	
		Distal DVT	Intermuscular DVT
1 h	15.2% (7/46)	4.3%(2/46)	10.9%(5/46)
6 h	8.7% (4/46)	0.0% (0/46)	8.7%(4/46)
12 h	15.6% (7/45)	2.2%(1/45)	13.3%(6/45)
18 h	17.8% (8/45)	2.2%(1/45)	15.6%(7/45)
20 h	21.7% (10/46)	6.5%(3/46)	15.2%(7/46)
Total	15.8% (36/228)	3.1%(7/228)	12.7%(29/228)
P	0.539	0.541	0.846

VTE: venous thromboembolism; DVT: deep vein thromboses

### Comparison of D-dimer and APTT AMONG the five groups

**Fig. 4:**
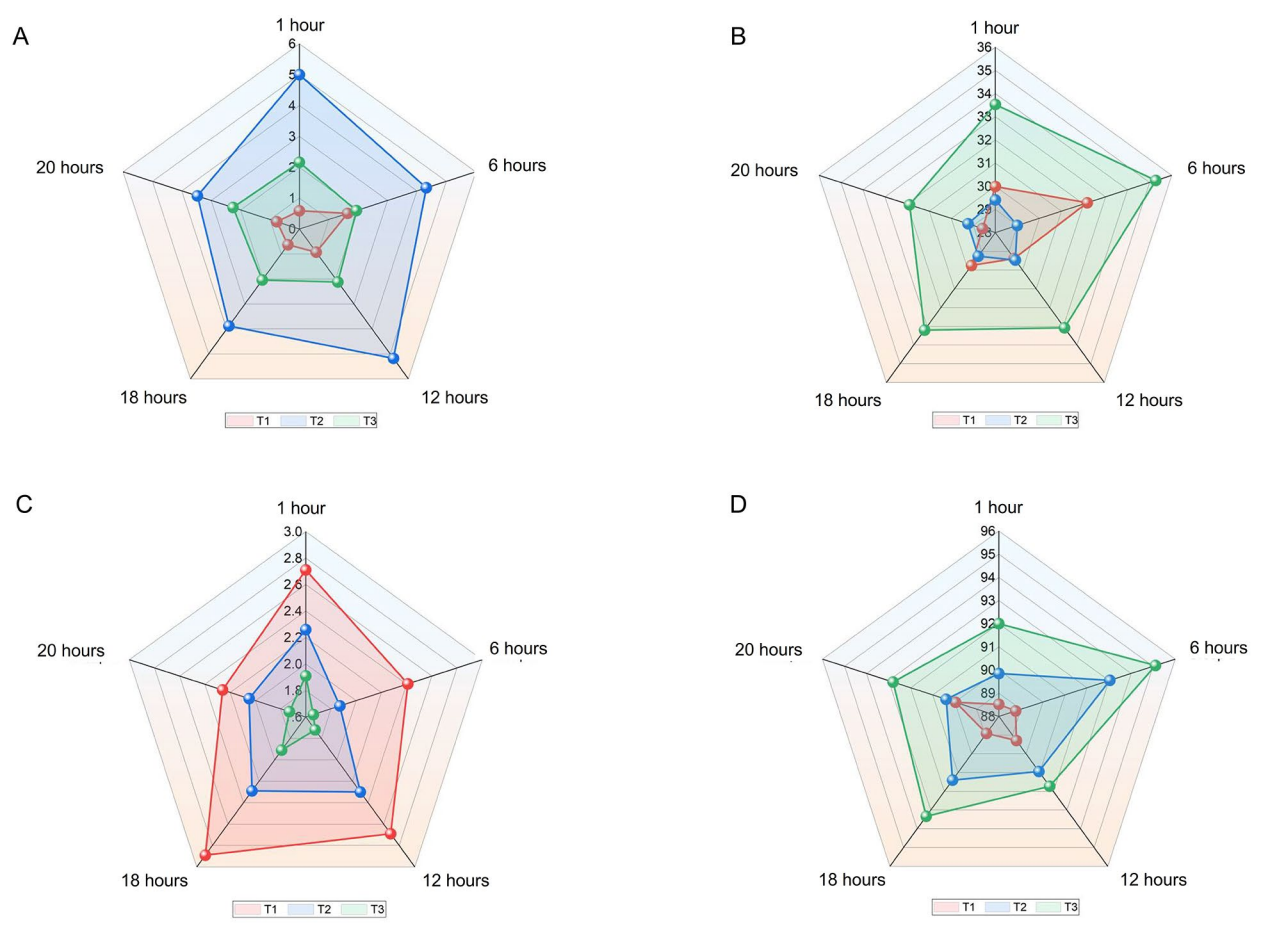
Radar chart of secondary outcomes in the five groups. **A.** D-dimer; **B.** Active partial thromboplastin time; **C.** Visual analogue scale; **D.** Comfort score

D-dimer significantly increased on a postoperative day 1 compared with that on preoperative 1 day, and it significantly decreased on postoperative day 3 among all five groups (*P* < 0.001, Table 3; Fig. 4A). Patients treated with 6-hour VFP had the lowest D-dimer among the five groups, but with no significant difference (*P* = 0.658). Patients treated with 6-hour VFP also had the highest APTT compared with that in the other groups (*P* = 0.262) at postoperative day 3 (Fig. 4B). The above results indicated that all five durations of VFP application showed favorable effects in the prevention of VTE.

**Table 3:** Comparison of D-dimer levels and APTT between the five groups before and after surgery

Groups	Patients	D-dimer (mg/L)			APTT (s)		
		Preoperative	Postoperative		Preoperative	Postoperative	
			1 day	3 days		1 day	3 days
1 hour	46	0.59 ± 0.82	5.00 ± 6.56	2.16 ± 2.72	29.99 ± 4.80	29.41 ± 4.56	33.53 ± 8.77
6 hours	46	1.64 ± 4.12	4.32 ± 4.71	1.94 ± 1.44	32.17 ± 5.28	29.00 ± 4.57	35.28 ± 10.10
12 hours	45	0.93 ± 1.25	5.18 ± 6.71	2.12 ± 1.51	29.38 ± 6.18	29.47 ± 5.89	33.08 ± 8.66
18 hours	45	0.63 ± 0.67	3.88 ± 4.65	2.04 ± 1.20	29.75 ± 5.17	29.26 ± 5.76	33.20 ± 9.14
20 hours	46	0.77 ± 1.64	3.47 ± 3.00	2.26 ± 1.27	28.58 ± 4.11	29.24 ± 5.35	31.89 ± 7.49
P values		P_a_=0.101, P_b_=0.658, P_c_<0.001			P_a_=0.467, P_b_=0.262, P_c_<0.001		

APTT: active partial thromboplastin time

P_a_ indicates interaction effect; P_b_ indicates effect between groups; P_c_ indicates time effect

### Comparison of pain and comfort outcomes among the five groups

The 6-hour VFP group had the lowest pain score (*P* = 0.206) and the highest comfort score (*P* = 0.288) compared with the other four groups, suggesting that this group had the optimal pain and comfort outcome. However, there was no significant difference. The pain score among five groups were decreased after the use of VFP, and the difference was significant (*P* < 0.001, Fig. 4C; Table 4). An inverse pattern was observed in terms of the comfort score (Fig. 4D).

**Table 4:** Comparison of pain and comfort scores between five groups before and after surgery

Groups	Patients	Pain score(VAS)			Comfort score		
		Preoperative	Postoperative		Preoperative	Postoperative	
			1 day	3 days		1 day	3 days
1 hour	46	2.71 ± 0.27	2.26 ± 0.18	1.91 ± 0.14	88.52 ± 1.54	89.85 ± 1.37	92.00 ± 1.18
6 hours	46	2.41 ± 0.27	1.87 ± 0.19	1.66 ± 0.15	88.76 ± 1.52	93.04 ± 1.37	95.11 ± 1.18
12 hours	45	2.69 ± 0.27	2.30 ± 0.18	1.72 ± 0.14	89.29 ± 1.56	90.94 ± 1.38	91.73 ± 1.19
18 hours	45	2.89 ± 0.27	2.29 ± 0.19	1.91 ± 0.15	88.91 ± 1.56	91.40 ± 1.38	93.33 ± 1.19
20 hours	46	2.26 ± 0.27	2.05 ± 0.18	1.73 ± 0.14	89.96 ± 1.54	90.39 ± 1.37	92.80 ± 1.18
P values		P_a_=0.638, P_b_=0.206, P_c_<0.001			P_a_=0.842, P_b_=0.288, P_c_<0.001		

VAS: visual analogue scale

P_a_ indicates interaction effec; P_b_ indicates effect between groups;P_c_ indicates time effect.

### Analysis of risk factors for predicting DVT

**Fig. 5:**
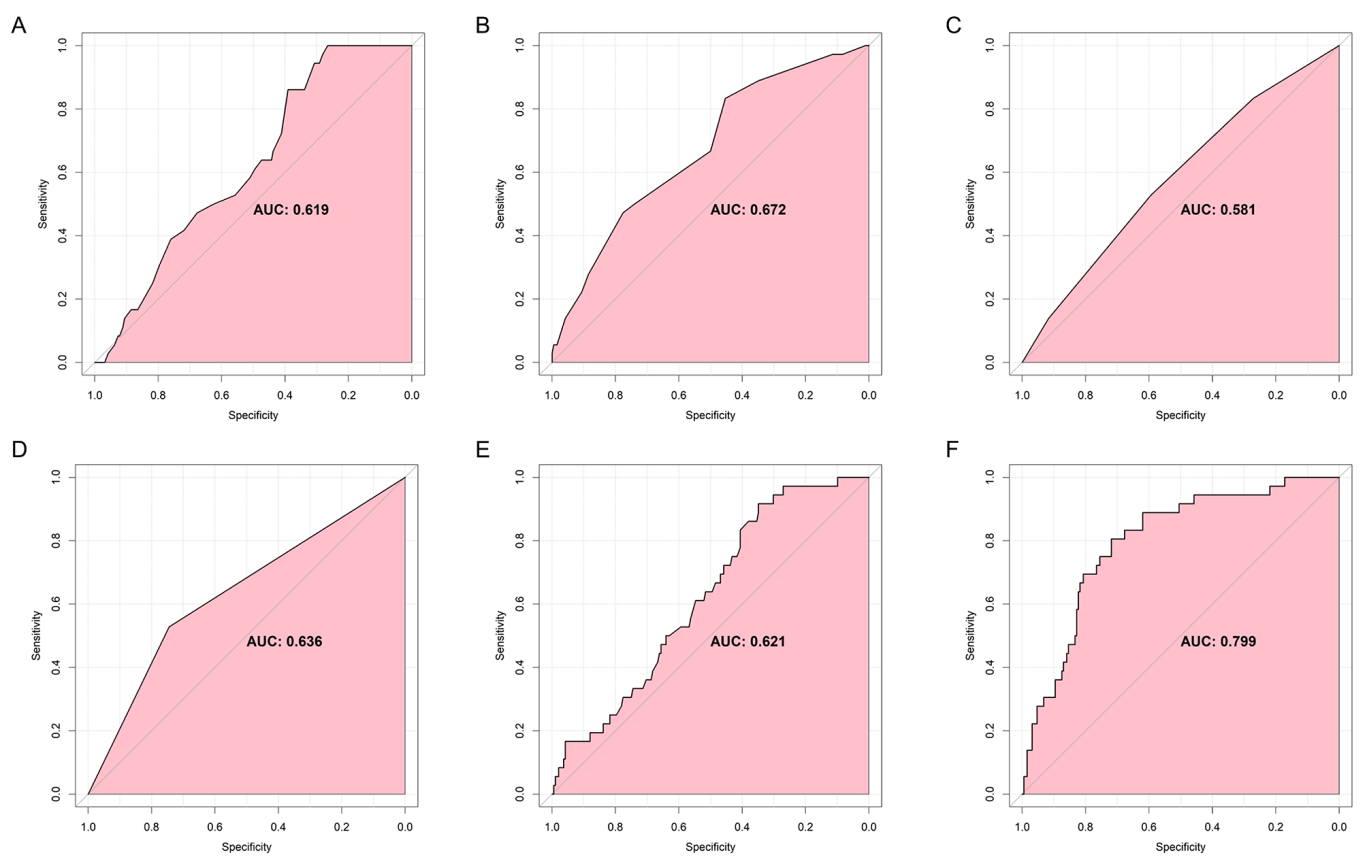
The area under the receiver operating characteristic curves for variables used to predict deep vein thrombosis. **A.** Age; **B.** Temperature; **C.** Blood type; **D.** Drainage; **E.** Platelet; **F.** The combination of all significant risk factors

Multivariate analysis revealed that older age (*P* = 0.036), higher temperature (*P* = 0.018), lower SBP (*P* = 0.016), AB (*P* = 0.046) or B (*P* = 0.021) blood type, drainage (*P* = 0.045), and lower platelet count (*P* = 0.047) were significantly positively associated with DVT (Table 5). The AUROC for age, temperature, blood type, drainage, platelet count, and the combination of all significant risk factors was 0.619, 0.672, 0.581, 0.636, 0.621, and 0.799, respectively (Fig. 5A-F). The above finding indicated that these variables were favorable predictors of DVT.

**Table 5:** Multivariate analysis of risk factors for predicting deep vein thromboses

Characteristics	OR	95% CI		P values
		LL	UL	
(Intercept)	0.003	0.000	1.888	0.079
Sex female	0.989	0.876	1.115	0.851
Age	1.005	1.000	1.010	0.036
Surgery left knee	0.985	0.860	1.129	0.830
Surgery right and left knee	0.872	0.677	1.124	0.293
Surgery right hip	1.053	0.904	1.227	0.505
Surgery left hip	1.094	0.948	1.263	0.222
Surgery right and left hip	0.938	0.666	1.320	0.714
Hypertension yes	1.055	0.936	1.188	0.382
Diabetes yes	1.005	0.685	1.475	0.978
Nationality minority	1.009	0.756	1.346	0.953
BMI	1.005	0.993	1.017	0.418
Temperature	1.232	1.037	1.464	0.018
Heart rate	0.994	0.987	1.002	0.124
Respiration rate	0.948	0.898	1.001	0.056
Systolic blood pressure	0.996	0.992	0.999	0.016
Diastolic blood pressure	1.001	0.995	1.007	0.734
Blood type AB	1.207	1.004	1.450	0.046
Blood type B	1.165	1.025	1.325	0.021
Blood type O	1.103	0.971	1.253	0.132
Anesthesia epidural	1.000	0.830	1.204	0.996
Blood loss	1.000	1.000	1.000	0.728
Operation time	1.000	0.999	1.001	0.642
Catheterization yes	0.956	0.452	2.025	0.907
Blood transfusion yes	0.987	0.861	1.130	0.845
Perioperative use of hemostatics yes	1.085	0.973	1.209	0.145
Drainage yes	1.127	1.003	1.265	0.045
Perioperative use of hormones yes	1.091	0.947	1.257	0.230
TT	1.011	0.984	1.040	0.418
APTT	0.995	0.982	1.007	0.386
PT	0.984	0.939	1.031	0.497
DD	0.979	0.955	1.005	0.115
Fibrin	0.986	0.963	1.010	0.251
Hemoglobin	1.000	0.997	1.004	0.841
Platelet count	0.999	0.998	1.000	0.047
ESR	1.001	0.996	1.007	0.582

OR: odds ratio; CI: confident interval; LL: lower limit; UP: upper limit; BMI: body mass index; TT: thrombin time; APTT: active partial thromboplastin time; PT: prothrombin time; ESR: erythrocyte sedimentation rate

## Discussion

### Main findings

The present study investigated the duration of applying VFP for the prevention of VTE in patients receiving hip and knee arthroplasty and found that six hours a day might be the optimal duration of using VFP in terms of VTE incidence, postoperative blood coagulation examinations, pain outcomes, and comfort scores. Patients treated with 6-hour VFP had the lowest incidence of DVT, lowest D-dimer, highest APTT, lowest pain score, and highest comfort score, compared with the other four groups, suggesting that 6-hour VFP was the most favorable duration of VFP application. Additionally, a series of risk factors for predicting DVT was identified in the study. To the best of our knowledge, this is the first study to explore the optimal duration of VFP application in patients undergoing hip and knee arthroplasty in a multicenter prospective clinical trial.

### Incidence of VTE in arthroplasty

Previous studies showed that the incidence of VTE after hip and knee arthroplasty was 20.6–58.2% [19]. However, the present study found that the incidence of VTE was 15.8%, and the distal DVT was only 3.1%. The incidence rate in our study was significantly lower than that reported by Itou et al. [20] and Kakkos et al. [21], who found that the incidence of VTE was 31.6% and the distal DVT was 9.28% after hip and knee arthroplasty when only pharmacologic thromboprophylaxis was performed for patients, respectively. These findings indicated that a combination of pharmacologic and mechanical thromboprophylaxis could further decrease the incidence of VTE, and its effect was superior to pharmacologic thromboprophylaxis alone.

In addition, our study showed that the D-dimer was decreased and APTT was increased after using VFP, and the difference was statistically significant (*P* < 0.001), which implied that the preventive strategy used in the current study could effectively improve the hypercoagulable state and slow down the coagulation speed. This conclusion is consistent with the findings of Sakai et al. [22].

### Duration of VFP application for prevention of VTE

At present, there is no consensus on the duration of VFP application to prevent VTE events in patients undergoing knee and hip orthopaedic surgery. Some studies have proposed the continuous application of 20-hour VFP, while others have recommended a 30-minute to 1-hour VFP application. Charalambous et al. [11] found that 20-hour VFP application reduced the incidence of VTE; however, patients in their study were bedridden, whereas patients in our study underwent routine out-of-bed activity on the second day after hip and knee arthroplasty, and were not suitable for continuous mechanical prophylaxis. Some previous studies were based on short-duration VFP application and failed to elaborate the basis for the selection of the duration of use, and therefore their conclusions were drawn only by comparing the effect of VFP application for 30 min/day to 3 h/day, which has some limitations in the study design. Conversely, the grouping scheme of our study has a comprehensive coverage of application duration, more targeted conclusions, and a multicenter, prospective randomized controlled trial design, which is the type with the highest evidence-based rating in methodological quality assessment, and the findings are worthy of consideration. Although no statistically significant difference was found in the incidence of VTE among the different duration groups, the rate was lowest in the 6-hour VFP group and highest in the 20-hour VFP group.

Comfort scores improved in all groups after VFP application, and pain scores gradually decreased, all with statistically significant differences (*P* < 0.05). The 6-hour/day group had the highest comfort scores and the lowest pain scores after 3 days of VFP application. There were discrepancies between patient feedback and the current literature on the evaluation of IPC. Pamela et al. [23] concluded that IPC wrapped from the foot to the thigh was bulky and noisy and patients felt constrained and had poor sleep quality and difficulty completing continuous mechanical prophylaxis. In contrast, during the implementation of the present study, patients in the > 18-hour/day group had better compliance, with 0 cases of patient loss due to device intolerance, and subjective evaluation of a good experience was perceived as more acceptable by the experimenter when communicating with the patients.

### Mechanism of using VFP for prevention of VTE

The mechanism of using VFP for the prevention of VTE involves venous hemodynamics of the lower extremity, which is mainly relevant to the physiology of the human plantar venous pump. Through pulsatile pressure release, VFP promotes venous blood return by rapidly squeezing blood from the plantar venous plexus back into the lower limbs [10]. Simultaneously, VFP intermittently inflates and pressurizes the bottom of the foot like a foot massage and promotes blood return to the plantar venous plexus, relieving fatigue and pain, which provides a reasonable explanation for the ability of VFP to improve patient comfort and sleep quality [24].

However, VFP has a minimal impact on the venous blood flow between the calf muscles, which might explain why the incidence of intermuscular DVT was 12.7% and the distal DVT was only 3.1% in the present study. Hence, additional interventions are recommended to promote blood return to intermuscular veins of the lower leg when using VFP. Ankle pump exercises as one of the commonly used basic prophylactic measures can activate the calf muscle pump, which accelerates venous blood flow in the lower limbs and alleviates blood stasis through regular contraction and relaxation occurring in the calf flounder and tibialis anterior muscles [25]. Therefore, when VFP is used as a mechanical prophylaxis after hip and knee arthroplasty, patients should be encouraged to actively perform ankle pump exercises for more comprehensive and effective prevention of VTE.

#### Risk factors for predicting DVT

Multivariate analysis demonstrated that older age, higher temperature, lower SBP, AB or B blood type, drainage, and lower platelet count were significantly positively associated with DVT. Previous studies found that older age [26], AB or B blood type [27], and drainage [28] were significant contributors to DVT, which is consistent with the findings of the present study. Moreover, our results are congruous with previous investigations that elevated body temperature may be caused by a series of inflammatory responses in the body that increase the risk of DVT in patients. We found that lower SBP was positively associated with an increased risk of DVT. Several studies reported no association between SBP and the risk of DVT [29, 30]. Contrarily, the Age and Thrombosis, Acquired and Genetic Risk Factors in the Elderly (AT-AGE) study and a meta-analysis also reported an inverse association between SBP and the risk of DVT [31, 32]. In a Mendelian randomization study, no association was found between SBP and the risk of DVT [33], which suggests that the association between blood pressure and DVT may be explained by unmeasured confounding.

We also found that patients with non-O blood types—especially type B or AB—were at a significantly higher risk for DVT. Several previous studies reported that individuals with non-O blood types (A, B, and AB) had an increased risk of VTEs [27, 34]. Similarly, Jared et al. [35] performed a retrospective survey and reported a higher frequency of AB blood type in 887 patients with DVT compared with 27,138 healthy blood donors (6 vs. 4.5%, *P* < 0.05). Increased levels of von Willebrand factor, factor VIII (FVIII), cholesterol, and several inflammatory cytokines could be the pathogenic mechanisms underlying the increased risk of VTE in individuals with non-O blood types (A, B, and AB) [27]. Furthermore, whether drains should be left in place after arthroplasty remains controversial. Although previous studies showed that the usage of drainage was not associated with DVT after total joint arthroplasty [36, 37], our study demonstrated that the use of drainage after total joint arthroplasty was positively associated with a significantly increased risk of DVT. Although patients can walk full weight-bearing the next day after arthroplasty with drains in situ, most patients worry about the risk of incarceration of knee flexion or hip joint during drainage, which undoubtedly limits early postoperative activities of patients, thus increasing the risk of DVT. While some studies found that platelet count was a risk factor for DVT, other studies noted that a lower platelet count was closely related to most DVTs [38–40]. We observed a significantly lower preoperative platelet count in patients with DVT. This may be because platelet count could reflect platelet consumption during venous thrombus formation, highlighting the relevant pathophysiological role of platelets during DVT.

### Limitations

This study has study several drawbacks. First, this study only investigated the early prognosis of applying VFP to prevent VTE; the follow-up was only three days after surgery, while relative long-term outcomes were not evaluated in the study. Second, this study did not assess the occurrence of complications such as skin pressure injuries, which is crucial to ensure the safety of VFP for the duration of application. Thus, validation studies are still warranted in the future. Finally, due to the small sample size of this study, no definite conclusion can be drawn. In the future, multi-center studies with large samples are needed to provide more robust evidence for the standardized application of plantar venous pumps in clinical practice.

## Conclusions

In summary, six hours a day may be the optimal duration of applying VFP for the prevention of VTE in patients receiving hip and knee arthroplasty. However, further studies are needed to validate these findings.

### Electronic supplementary material

Below is the link to the electronic supplementary material.

Supplementary Material 1

## Data Availability

Data and materials are available upon reasonable request with the permission of the corresponding authors.
