# Supplementary material for: Effect of different application duration of a venous foot pump on prevention of venous thromboembolism after hip and knee arthroplasty: a multicenter prospective clinical trial
Source: BMC Musculoskelet Disord. 2023 Dec 1;24:931. doi: 10.1186/s12891-023-06921-w (PMC10691185; doi:10.1186/s12891-023-06921-w)
Supplement: Supplementary file 1 — Supplementary Material 1 [file 12891_2023_6921_MOESM1_ESM.docx]

Supplementary figure s1. Consort Diagram: Flow of the participants throughout the study
